# Differential Gene Expression Analysis of Placentas with Increased Vascular Resistance and Pre-Eclampsia Using Whole-Genome Microarrays

**DOI:** 10.1155/2011/472354

**Published:** 2011-03-08

**Authors:** M. Centlow, C. Wingren, C. Borrebaeck, M. J. Brownstein, S. R. Hansson

**Affiliations:** ^1^Departments of Obstetrics and Gynecology and Clinical Sciences, Lund University, BMC C14, Klinikgatan 28, 221 84 Lund, Sweden; ^2^Department of Immunotechnology and CREATE Health, Lund University, 22100 Lund, Sweden; ^3^National Institute of Mental Health, NIH, Bethesda, MD 20892-9663, USA

## Abstract

Pre-eclampsia is a pregnancy complication characterized by hypertension and proteinuria. There are several factors associated with an increased risk of developing pre-eclampsia, one of which is increased uterine artery resistance, referred to as “notching”. However, some women do not progress into pre-eclampsia whereas others may have a higher risk of doing so. The placenta, central in pre-eclampsia pathology, may express genes associated with either protection or progression into pre-eclampsia. In order to search for genes associated with protection or progression, whole-genome profiling was performed. Placental tissue from 15 controls, 10 pre-eclamptic, 5 pre-eclampsia with notching, and 5 with notching only were analyzed using microarray and antibody microarrays to study some of the same gene product and functionally related ones. The microarray showed 148 genes to be significantly altered between the four groups. In the preeclamptic group compared to notch only, there was increased expression of genes related to chemotaxis and the NF-kappa B pathway and decreased expression of genes related to antigen processing and presentation, such as human leukocyte antigen B. Our results indicate that progression of pre-eclampsia from notching may involve the development of inflammation. Increased expression of antigen-presenting genes, as seen in the notch-only placenta, may prevent this inflammatory response and, thereby, protect the patient from developing pre-eclampsia.

## 1. Introduction

In the western world, 3–7% of all pregnancies are affected by pre-eclampsia (PE). The etiology of PE is still poorly understood, and several theories have been put forward [1–4]. 

PE is considered a two-stage disease [5, 6]. The first stage is characterized by poor placentation resulting from shallow invasion of the trophoblasts into the maternal spiral arteries. Instead of the low-resistance high-flow system seen in the normal placenta, the PE placenta has increased resistance, and a decreased blood flow and uneven perfusion leads to hypoxia, ischemia, and reperfusion injuries in the placenta [5, 6]. 

The second stage of PE is characterized by a general vascular endothelial damage, which eventually affects all maternal organs. Transition from stage one, to stage two of the disease is typically seen before 35 gestational weeks (GW) in early onset PE. Placentas from early onset PE more often show histological pathological findings associated with poor placental perfusion, such as infarcts and chronic inflammation [7]. In contrast, placentas from late-onset PE, manifest after 35 GW, rarely show these pathological findings. The placental factors linking the first and second stage are not fully known. However, some placental factors that may participate in the progression to the second stage have been identified [8]. 

Increased vascular resistance in the uterine arteries is associated with decreased placental perfusion. The former can be measured by Doppler ultrasound as early as 12 GW and quantified using pulsatility index (PI) which is calculated by subtracting the peak systolic velocity from the end diastolic velocity and dividing the difference by the maximum velocity averaged over one cardiac cycle. Increasing PI suggests increased vascular resistance, which has been shown to correlate well with early onset and severe PE [9]. Increased uterine artery resistance is also correlated with a diastolic pattern described as “notching” detected in early pregnancy [10]. As many as 15–20% of all pregnancies show signs of bilateral notch at 18 GW, although this number decreases to about 5% when patients are re-evaluated at 24 GW [11]. The presence of bilateral notch has been shown to be a predictor of early onset PE [12]. Although persisting notching is a predictor of women with increased risk of developing PE later in their pregnancies, not all of them do [9, 13, 14]. Thus it seems possible that women with notching, who do not develop PE later in their pregnancies, may be protected from doing so by mechanisms that could include expression of beneficial genes or inhibition of the expression of harmful ones in their placentas. 

In a previous study of a small group of candidate genes, we provided evidence that notching may be an early stage of PE [15]. In the present study, we have used a comprehensive whole-genome oligonucleotide array system to profile the placental gene expression in two types of notch patients and PE pregnancy placentas to search for placental genes or groups of genes that might alter the progression to PE.

## 2. Material and Methods

### 2.1. Sample Collection

Placental samples were obtained from 35 women admitted to Lund University Hospital (15 controls, 10 PE without notch, 5 PE with notches, and 5 notches without PE, Table 1). This, sample set that we studied earlier [16]. Sampling was performed after written consent and the approval by the Swedish Ethical Committee Review Board were given. Pre-eclampsia was defined as blood pressure above 140/90 mmHg and proteinuria above 0.3 g/l [17]. Uterine artery Doppler velocimetry was performed at 18 and 24 GW, using a Philips HDI 5000 system (Philips Medical Systems, Vothell, USA). The uterine artery was visualized with color Doppler imaging on each side at the level of crossing with the external iliac artery. The pulsatility index (PI) was computed automatically by the system in both the right and left uterine arteries. The presence or absence of a diastolic notch in the waveform was assessed visually. Only patients with bilateral notching were included in the notch group. A 10 × 10 × 10 mm cube of villous tissue from one cotyledon was removed after delivery and immediately put on dry ice. Since the placenta is a very heterogeneous tissue and the placenta gene expression differs between different parts of the placenta, sampling was always performed in a central portion of the placenta in an attempt to keep the tissue samples as homogenous as possible [18]. Maternal blood samples, 6 ml, were collected before delivery into PAXgene blood RNA tubes (Qiagen, Solna, Sweden) according to the manufacturer's instructions. Samples were stored at −80°C until usage.

### 2.2. RNA Extraction

#### 2.2.1. Tissue

RNA was extracted with TRIzol according to the manufacturer's instructions (Invitrogen, Carlsbad, USA). Briefly, 120–150 mg of placental tissue was homogenized in 2-3 ml TRIzol on ice. Cellular debris was removed by spinning the samples at 12000 g for 10 minutes after which chloroform was added. The aqueous phase containing the RNA was isolated. To precipitate the RNA, 2-propanol with sodium citrate was used. The sodium citrate was added in order to remove contaminating proteoglycans and polysaccharides. The RNA pellet was washed once with 75% (v/v) ethanol, and then allowed to air dry. The pellet was dissolved in RNase-free water and assayed using a NanoDrop spectrophotometer (Thermo Fisher Scientific, Waltham, USA). To remove residual phenol, the RNA was again precipitated with 2-propanol, and washed three times with 75% (v/v) ethanol before it was used. 

RNA integrity was assessed on a 1% (w/v) agarose gel containing 6.7% (v/v) formaldehyde run with 1 × MOPS buffer. RNA samples were required to have clear bands at 28S and 18S, respectively, with no other bands present on the gel. No samples with poor RNA quality were used for further analysis.

#### 2.2.2. Blood

RNA was extracted using PAXgene Blood RNA Kit according to the manufacturer's instructions (Qiagen). Briefly, samples were centrifuged and the supernatant discarded after which the pellet was resuspended in 5 ml RNase-free water. Following centrifugation, proteins were removed by the addition of proteinase K. Samples were added to PAXgene columns and washed with the corresponding buffers after which RNA was eluted in 80 *μ*l elution buffer. RNA was quantified and assessed as above.

### 2.3. DNA Microarrays

Microarrays were produced at the Swegene DNA Microarray Resource Center, Department of Oncology, Lund University, Sweden (http://swegene.onk.lu.se). Human array-ready oligonucleotide libraries Version 2.1 and Version 2.1.1-upgrade, comprising approximately 27, 000 unique probes, were obtained from Operon (Operon Biotechnologies, Germany). Probes were dissolved in Corning Universal Spotting solution (Corning, Acton, MA) and printed on aminosilane coated glass slides (Corning) using a MicroGrid2 robot (BioRobotics, Cambridgeshire, UK) equipped with MicroSpot 10K pins (BioRobotics, Cambridgeshire, UK). Following printing, arrays were left in a desiccator to dry for 48 hours, rehydrated for 1 second over steaming water, snap-dried on a hot plate (98°C), and UV cross-linked (800 mJ/cm^2^).

Complementary DNA for microarray was synthesized and labeled with fluorescence, Cy3 or Cy5 (Amersham Biosciences, Buckinghamshire, UK), using the Promega ChipShot labeling system according to the manufacturers' instructions (Corning). Briefly, Cy3- and Cy5-labeled cDNA was prepared using 5 *μ*g total RNA. Pretreatment of arrays, hybridization, and posthybridization wash carried out according to the manufacturers' instructions (Promega ChipShot labeling system and Promega, Madison, USA). All experimental samples were hybridized individually against a common reference (a placental sample from a normotensive pregnancy without any complications). Samples were run in single experiments. 

Samples were run individually (i.e., no groups were pooled). The arrays were not run in replicates, each sample was only assessed once. 

#### 2.3.1. Imaging and Data Analysis

To image hybridized microarray slides, an Agilent G2565AA microarray scanner (Agilent Technologies, Palo Alto, USA) was used. Image analysis was done in GenePix Pro 4.0 (Axon Instruments Inc., Foster City, USA). Fluorescence intensities were extracted and spots with grains of dust and other contaminants were identified and eliminated. The retrieved intensity values were uploaded into Bio Array Software Environment (BASE) (http://base.thep.lu.se) for further analysis [19]. In BASE, Cy3 and Cy5 signals were corrected for background using the median spot pixel intensity and median local background pixel intensity. Fluorescence ratios were calculated as Cy3/Cy5 and log (base 2) transformed. Spots were filtered for intensity below 350 in any of the two channels and biased spots identified in Genepix. Following that, filtering spots were normalized using Lowess normalization and then corrected for common reference bias using gene median centering, after which the data was filtered for fold change equal to or above 1.5. [20]. Group comparison was conducted using a false discovery rate modified *t*-test [21]. A *P*-value <.005 was considered statistically significant. The data has been uploaded by MIAMExpress submission and annotation tool European Molecular Biology Laboratory European Bioinformatic Institute Microarray Informatics.

#### 2.3.2. Bioinformatic Analysis

We used Gene Ontology (GO, http://www.geneontology.org/), the Kyoto Encyclopedia of Genes and Genomes (KEGG, http://www.genome.jp/kegg/), and InterPro (http://www.ebi.ac.uk/interPro/) to classify significantly altered genes according to their molecular function, their participation in signaling pathways, and the presence of protein domains, respectively, as previously described [15]. The genes were also annotated and analyzed using the database for annotation, visualization, and integrated discovery (DAVID) with the whole human genome, approximately 27,000 genes, as background [22]. Statistical differences in GO or KEGG were determined by comparing the expected number of genes in each GO-category/KEGG-pathway (as determined by the background genome) with the actual number of altered genes as determined by the microarray results. In each category, both activating and suppressing genes may be present. Altered categories as well as the genes corresponding to them are listed in Table 3. A *P*-value below  .05 with a fold change above 2 was used as cutoff.

### 2.4. Quantitative Real-Time PCR

#### 2.4.1. cDNA Synthesis

cDNA was synthesized from total RNA using reverse transcriptase according to the manufacturer's instructions (Applied Biosystems, Foster City, USA) as previously described [16]. cDNA samples were stored in −20°C until usage.

#### 2.4.2. Real-Time Assays

Taqman Gene Expression Assays were ordered from Assays-on-Demand (*TGFB1*: Hs00171257_m1, *HP*: Hs00978377_m1, *TIP30*: Hs00185131_m1 and *INHA*: Hs00171410_m1; Applied Biosystems, Foster City, USA). Primers were designed to target exon boundaries in order to avoid amplifying genomic DNA. Transcripts were assayed using an ABI Prism 7000 sequence detection system and quantified by using a standard curve by means of a 4-fold dilution series (80–0.08 ng). Each sample was run individually, and the reactions were run in duplicates as previously described [16]. Samples were removed from statistical analysis if only one of the two reactions gave a reliable result or if the standard deviation (SD) between the duplicates was above 2SD.

#### 2.4.3. Analysis

**β**-actin was used to normalize the real-time PCR data. A Kruskal-Wallis test was used to determine the statistical distribution between the four groups (PE, PE with notching, PE without notching, notching without PE, and controls). If the distribution was significant, a post hoc Dunn's multiple comparison test was used to determine significance between individual groups. A *P*-value <.05 was considered statistically significant.

### 2.5. Antibody Microarrays

An in-house designed human recombinant antibody microarray technology platform was applied for targeted protein expression profiling [23, 24]. The applicability of the technology platform has been extensively validated [23–26] and demonstrated in various clinical applications targeting, for example, pre-eclampsia [27] and pancreatic cancer [28].

#### 2.5.1. Extraction and Labeling of Placenta Tissue Proteins

Predominantly water-soluble proteins were extracted from tissue biopsies as recently described [27]. Briefly, 50 mg tissue was resuspended in 375 *μ*l extraction buffer—2% (w/v) saponin (Sigma-Aldrich, St Louis, MO, USA), 100 *μ*g/ml soybean trypsin inhibitor (Sigma-Aldrich), 350 *μ*g/ml phenylmethylsulfonyl-fluoride (Sigma-Aldrich), and 0.1% (w/v) bovine serum albumin in PBS—and incubated at 4°C over night. After centrifugation at 13000 g for 5 min, the supernatants containing the extracted proteins were collected and dialyzed against PBS for 72 h at 4°C. The protein concentration was determined using Micro BCA Protein Assay kit (Thermo Fisher Scientific Pierce, Rockford, IL, USA) according to the instructions provided by the manufacturer. 

The samples were biotinylated, using previously optimized labeling conditions [23, 24, 29]. Briefly, the samples were diluted with PBS to a final protein concentration of about 2 mg/ml before EZ-Link Sulfo-NHS-LC-Biotin (Thermo Fisher Scientific Pierce) was added to a final concentration of 0.6 mM. The reaction mixture was then incubated for 2 h on ice, with vortexing every 20 min. Unreacted biotin was removed by dialysis against PBS for 72 h at 4°C. Finally, the samples were aliquoted and stored at −20°C prior to use.

#### 2.5.2. Production and Purification of scFvs

Eleven human recombinant scFv antibodies against 4 soluble proteins, including, TGF-*β*1 (clones 1 to 3), VEGF (clones 1 to 4), C5 (clones 1 and 2), and RANTES (clones 1 and 2), were included in the study. Further, 22 additional antibodies directed against a range of analytes were included for array-to-array normalization (see below). The antibodies were selected from the n-CoDeR library [30] and kindly provided by BioInvent International AB (Lund, Sweden). Thus, some of the antigens were recognized by up to four different scFv clones, which was part of the quality control of the arrays. 

All scFvs were produced in E. coli. Briefly, soluble scFvs, all carrying a C-terminal his6-tag, were purified from expression supernatants or periplasmic space by affinity chromatography on Ni^2+^-NTA (Qiagen, Hilden, Germany). Bound molecules were eluted with 250 mM imidazole, dialyzed against PBS, and stored at 4°C until further use. The integrity and degree of purity of the produced scFvs were evaluated by 10% SDS-PAGE (Invitrogen, Carlsbad, CA, USA). The protein concentrations were determined by measuring the absorbance at 280 nm.

#### 2.5.3. Fabrication and Processing of scFv Antibody Microarrays

The fabrication and processing of scFv microarrays were performed according to previously optimized techniques [27]. Briefly, scFv antibody microarrays were fabricated by dispensing Ni^2+^-NTA purified scFvs (200–500 *μ*g/ml) onto black polymer MaxiSorp slides (NUNC A/S) using the noncontact sciFLEXARRAYER S11 (Scienion AG, Berlin, Germany). The scFvs were arrayed, in eight replicates each, by spotting 2 drops (300 pL/drop) on top of each other (the spots were allowed to dry out in between), and individual subarrays were created using a hydrophobic marker pen (DakoCytomation). 

A 36 × 8 array, composed of 33 antibodies, 1 negative control (PBS), and 2 position markers (10 *μ*g/ml Alexa-647 labeled streptavidin in PBS) was printed per slide. The arrays were blocked with 5% (w/v) fat-free milk powder and 0.05% (v/v) Tween-20 in PBS for 1 h on shaking table and then washed two times with 0.05% (v/v) Tween-20 in PBS (PBS-T). All incubation steps were performed in humidity chamber unless otherwise stated. Labeled tissue extracts diluted 10 times in 1% (w/v) fat-free milk powder and 1% Tween-20 in PBS (PBS-MT) were added to arrays (100 *μ*l/slide) and incubated on a shakingtable for 1 h. Subsequently, the arrays were washed two times with PBS-T. Bound proteins were detected using 1 *μ*g/ml Alexa-647 labeled streptavidin in PBS-MT, and incubated on a shakingtable for 1 h. The slides were washed two times with PBS-T. Finally, the arrays were dried under a stream of nitrogen and immediately scanned at 5 *μ*m resolution using a confocal fluorescence scanner (ScanArray Express microarray scanner; Perkin Elmer Life Analytical Sciences). The intensity of each spot was quantified by the fixed circle method using the ScanArray Express software V4.0 (Perkin Elmer Life Analytical Sciences). Each data point represents the mean value of four replicates (the two highest and two lowest replicates were automatically excluded) after subtracting local background. For protein analytes displaying saturated signals, values from lower scanning settings were scaled and used instead.

#### 2.5.4. Data Normalization

The chip-to-chip normalization was performed using a semiglobal normalization approach, as previously described [30, 31]. To this end, 22 antibodies were used to calculate a chip-to-chip normalization factor. The normalization factor Ni was calculated by the formula Ni=Si/u, where Si is the sum of the signal intensities of the 22 analytes for each sample, and u is the average Si from all samples. Each dataset generated from one sample was divided by normalization factor Ni.

#### 2.5.5. Data Analysis

Significantly upregulated or downregulated analytes (*P* < .05) were identified using Wilcoxon test using R [32].

## 3. Results

We compared the gene profiles of PE placentas to those of normal placentas using oligonucleotide microarrays. A total of 35 samples were included in the experiments, 10 PE placenta samples, 15 control placenta samples, 5 placenta samples from patients with bilateral notching with PE, and 5 placenta samples from patients with bilateral notching without PE (Table 1). In order to identify genes differentially expressed between the examined groups, we performed a false discovery rate modified *t*-test. To limit the number of significant genes a cutoff of value was set, *P* ≤ 10^−3^. At this *P*-value, 148 genes were significantly altered in at least one group comparison. The differentially expressed genes are listed in the supplementary table (see supplementary Material available onlinr at doi: 10.155/2011/472354). Among the most significantly altered genes were neural cell adhesion molecule 1 (*NCAM1*), haptoglobin (*HP*), transforming growth factor beta 1 (*TGFB1*), and inhibin alpha (*INHA*) (Table 2).

In order to assess the possibility that the results obtained derived from maternal blood in the placentas, 10 maternal blood samples (5 PE and 5 controls) were analyzed.

Using the same *P*-value as above, resulted in 10 genes with significantly altered expression between PE and controls; actin-related protein 2 (*ACTR2*), calponin 2 (*CALP2*), and superoxide dismutase 2 (*SOD2*). None of theses genes matched the altered placenta genes indicating that maternal blood trapped in the placenta did not affect the results observed there.

**Figure 1:**
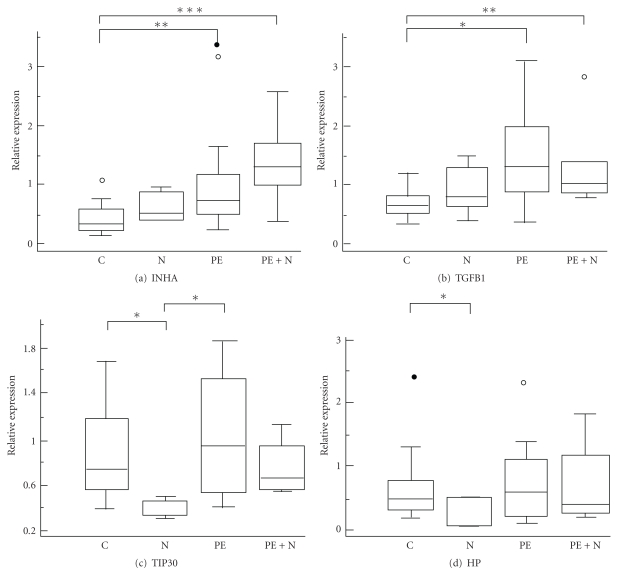
Microarray validation with real-time PCR. The most significantly altered genes in the microarray analysis as well as genes of interest were validated using quantitative real-time PCR. Results are presented as box-plots, showing groups' medians as well as the 25th and the 75th percentile. *β*-actin was used as housekeeping gene, and all values are quotas between the gene of interest and *β*-actin. Kruskal-Wallis with post hoc Dunn was used to determine statistical significance. *P < .05, **P < .01, ***P < .005 The order is as follows: (a) inhibin *α*, (b) transforming growth factor *β*, (c) Tat-interacting protein (30 kD), and (d) haptoglobin.

Quantitative real-time PCR was used to verify the gene microarray results. Real-time mRNA data are presented as box-plots in Figure 1.

**PE Compared to Controls**

*TGFB1* was higher in the PE with and without bilateral notching than in the controls (*P* = .02 and *P* = .01, resp.). *INHA* showed increased expression in the PE groups (with and without notching) versus controls (*P* = .005 and .01, resp.).

**PE Compared to Notch without PE**

the transcription factor Tat-interacting protein, 30 kD, (*TIP30*) was underexpressed in both the notch groups compared to the PE group (*P* = .02) and controls (*P* = .04). Neural cell adhesion molecule 1 (*NCAM1*) was not significantly altered in the real-timePCR analysis. A larger sample size will be needed to validate or invalidate the *NCAM *result definitively.

**Notch Compared to Controls**

haptoglobin (*HP*), a plasma heme scavenger responsible for transporting heme to the liver, showed lower expression in the notch group compared to controls (*P* = .04).

To couple changes in gene expression to biological processes, we performed three different bioinformatic analyses: gene ontology (GO), KEGG signaling pathway analysis, and InterPro protein domain analysis (IRP). Results from the GO and KEGG/IRP analyses are listed in Tables 3 and 4, respectively.

When comparing genes differentially expressed in the PE group against controls, 18 GO categories were significantly altered *P* < .05; the most significant changes were in genes associated with transcription factor binding (GO:0008134, *P* = .0019), negative regulation of signal transduction (GO:0009968, *P* = .0027), and transcription corepressor activity (GO:0003714, *P* = .0086). When performing a signal pathway analysis, of the same genes, the neurodegenerative disorders pathway (KEGG pathway ID HSA01510, *P* = .04) was the only one significantly altered. This pathway includes genes such as heat shock protein 5 and superoxide dismutase 2. In the InterPro analysis, the category Ras small GTPase, Rab (IPR003579) type was the only category altered (*P* = .05).

When comparing PE to the notch group, 36 GO categories were found to be altered, although several of these were “hierarchical duplicates,” meaning they were subgroups within the same categorical branch. Again, the top categories were transcription related, transcriptional activator activity (GO:0016563, *P* = .0005), and transcription coactivator activity (GO:0003713, *P* = .0009). Muscle fiber development was also significantly altered (GO:0048747, *P* = .0058). The signaling pathway antigen processing and presenting (KEGG pathway ID HSA04612, *P* = .045) showed a significant alteration. In the InterPro analysis, six protein domain functions were altered. Among them, were the HEAT repeat element (IPR000357, *P* = .0028) and H+ transporting ATPase, proton pump (IPR000695, *P* = .0078).

In the comparison between PE with notching and notch without PE, 19 GO categories were altered. Among the most significantly altered categories were chemotaxis (GO:0006935, *P* = .021), positive regulation of cell proliferation (GO:0008284, *P* = .026), and cytokine binding (GO:0042102, *P* = .048). The ubiquitin mediated proteolysis signaling pathway (KEGG pathway ID HSA04120, *P* = .02) was altered. The InterPro analysis showed changes in ATPase protein domains (IPR002379, *P* = .02).

To validate a selected differentially expressed gene, TGF-b1, on the protein level, a recombinant antibody microarray technology platform, optimized for sensitive serum protein expression profiling, was applied. For this purpose, we selected 6 PE and 5 control samples to assay. Three additional proteins commonly involved in inflammatory responses—C5, VEGF, and RANTES—were also analyzed. It is worth noting that the mRNA encoding the VEGF receptor, FLT1, was found to be increased in PE samples in our DNA array study. 

**Figure 2:**
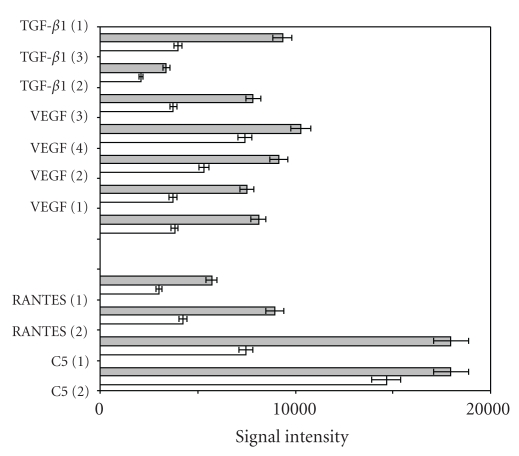
Protein expression validation with antibody microarray. Protein expression profiling of PE (grey bars) versus N (open bars) using recombinant antibody microarray analysis. A focused microarray composed of 11 scFv antibodies directed against 4 proteins, including TGF-*β*1, VEGF, C5, and RANTES was applied. Six PE samples and five control samples were analysed. The index (1), and so forth, indicate the clone number of the antibody used, meaning that several clones targeting different epitopes on the same analyte was used to further strengthen the data. The expression levels between PE and N were found to be significant (*P* < .05) for all four analytes.

TGF-*β*1, VEGF, C5, and RANTES were significantly upregulated in PE compared to controls, (*P* < .05) (Figure 2). Taken together, the data show that TGF-*β*1 was differentially expressed on both gene and protein level. Like its receptor, VEGF protein was upregulated leveling PE samples (FLT1, Table 2).

## 4. Discussion

To find genes or mechanisms that might promote or mitigate the development of PE, we used gene profiling to study women with bilateral notching who developed or failed to develop PE. Some of the altered genes (*INHA*, *FLT1*, *HO-1,* and *NCAM-1*) have been reported in other studies [8, 33, 34]. The V-rel reticuloendotheliosis viral oncogene homolog A (*RELA*) has previously been implicated in PE and, we now show that *RELB* is increased in PE as well [15]. A few HLA isoforms, HLA-G3, and HLA-DRA have previously been shown to be either increased or decreased in PE [34, 35].

**Figure 3:**
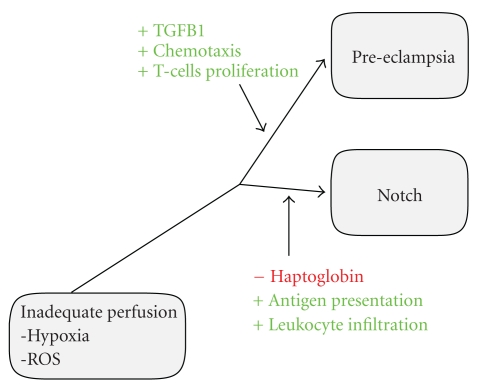
Pathophysiology. Based on the gene expression results, we suggest that there may be a placental mechanism that determines how PE progresses from stage one, characterized by inadequate perfusion of the placenta, to the clinical symptoms in stage two. Depending on which inflammatory genes that are expressed, the placenta may either progress from a state of notch to early onset PE or remain clinically asymptomatic only showing signs of bilateral notch.

Notching in early pregnancy is considered a risk factor for PE [9, 14]. We have previously suggested notch to be an early form of PE [15]. Thus, one would expect the notch group to have a different placental gene profile compared to the control and PE groups. Indeed, our bioinformatic analyses, as well as our previous study, show alterations in several functional categories for the notch group. Based on the gene expression results, we suggest that there may be a placental mechanism that determines how PE progresses (Figure 3). Depending on which inflammatory genes are being expressed, the placenta may either progress from a state of notch to early onset PE or remain clinically asymptomatic only showing signs of bilateral notch. One mechanism that provides protection may be the increased capacity for antigen presentation. Overexpression of HLA genes such as *HLA-B*, *CD74*, *PSME1,* and *PSME2,* may inhibit the inflammatory processes and thereby prevent progression of PE. Recently, we have shown that *HLA-DPA1* is increased in the notch placenta [16]. 

Increased gene expression and accumulation of free fetal hemoglobin have also been shown in the PE placentas [16]. Hemoglobin, in its free form, disrupts cell membranes causing cytolysis and formation of reactive oxidative species (ROS) [36, 37]. Moreover, the hemoglobin metabolites, heme and iron, are both potent redox agents disrupting cell membranes and membrane proteins by cytolysis. Due to its lipophilic nature, heme crosses cell membranes and damages cytosolic proteins, organelles, and DNA [38]. Furthermore, heme is a proinflammatory substance that stimulates both neutrophil activation and migration [39, 40]. There is an increased number of fetal cells, cell debris, and free fetal hemoglobin crossing over the damaged blood-placenta barrier into the maternal system [41, 42]. The notch placenta shows similar vascular damage as in PE. Fetal cells and cell debris may, therefore, also leak into the maternal circulation in the notch placenta. Increased expression of HLA-genes and other genes responsible for antigen processing may result in an increased immune defense in the notch placenta. Hence, notch placentas may not develop the same inflammatory response as seen in notch with PE and PE. In fact, genes related to inflammation were unaltered in the notch placenta. 

When comparing PE with and without notch to notch without PE, we found pro-inflammatory genes with increased expression, *TGFB1*, *INHA*, and chemokine ligand 8 (*CCL8*). Among the most significantly altered GO functional categories are chemotaxis and locomotory behavior. Thus, PE with notching appears to be associated with increased cellular movement compared to notching only. More specifically, the mode of cellular movement appears to be induced by genes related to chemotaxis that were overexpressed. The GO functional category cytokine binding (GO:0019955) was also upregulated, suggesting that a cytokine gradient may be responsible for the chemotaxis in PE with notching. Immunologically derived cells proliferate in response to cytokine gradients. In fact, T-cell proliferation was yet another category altered in the comparison between PE with notch and notch without PE. The progression from notch to PE may, therefore, be driven by induction of inflammation within the placenta. PE is known to be associated with increased levels of several pro-inflammatory cytokines, including interleukins −6, −8, and the tumor necrosis factor *α* (TNF*α*) [43, 44]. Moreover, genes related to the central inflammatory NF-kappa B pathway are altered in the PE placentas, which may explain the increase of pro-inflammatory factors in PE [15, 45]. Our results suggest that the notch state may be more than a risk factor, it may be a stepping stone towards PE, bridging an early decrease in placental perfusion with the final inflammatory steps needed to progress into the second stage of PE. By using the antibody array methodology, we have been able to display different inflammatory signatures between early and late-onset PE, further underlining the importance of inflammation in the PE pathogenesis [46].

Several confounding factors may contribute to the gene expression revealed by microarrays. Gestational age and mode of delivery are such factors. A placenta delivered at 35 gw differs from a term placenta. Due to the fact that there is no treatment for PE, the gestational ages of the included PE cases were generally lower than those of the controls. Although we aimed to match the groups based on gestational age, one early onset PE case was included in the PE group. Placentas from vaginal deliveries have been shown to have increased activity in the pro-inflammatory pathway NF-kappa B as well as increased levels of pro-inflammatory cytokines compared to placentas from Caesarian sections [47]. Hence, mode of delivery may well affect the gene expression and contribute to some of the changes seen in the study.

In this discovery study, we have performed gene expression analysis on both placenta and maternal blood, and validated targeted analytes on the protein level as well, to further enhance our fundamental knowledge of PE. Although these findings need to be validated and extended analysing novel, independent cohorts containing larger numbers of samples, the work has provided a clue about the pathophysiology of PE. Pro-inflammatory genes may drive the placenta towards PE, and protective genes may have the opposite effect. More specifically, the first stage, poor placentation, induces ROS formation and damage to the blood-placenta barrier as previously suggested [6]. A possible intermediate step may be brought on by increased apoptosis, activation of inflammatory pathways, imbalance in antioxidation, and increased vascular resistance as seen in bilateral notch. As the placenta attracts and/or activates immune cells, inflammation and irreversible damage to the blood-placenta barrier may occur. Increased, expression of HLA and antigen-presenting genes may ameliorate the inflammatory response.

## Supplementary Material

Supplementary table 1. Gene expression results from the microarray analysis. A matrix showing all genes that were found to be significantly altered. Results are presented as P-value (Fold change). Genes with a p-value<0.005 and FC>1.5 were considered statistically significant. PE=pre-eclampsia, N=bilateral notching without pre-clampsia and PEwN=preeclampsia with bilateral notching.

## Figures and Tables

**Table 1:** Clinical characteristics of patients at delivery.

	Control	PE	PE/N	Notch
*N*	15	10	5	5
Age (y)	31 (24–37)	29 (22–34)	32 (27–39)	32 (23–39)
Gestational age (days)	280 (253–295)	264 (227–285)	241 (204–283)	250 (219–284)
Systolic pressure (mmHg)	120 (110–135)	159 (140–170)	151 (145–160)	110 (115–120)
Diastolic pressure (mmHg)	75 (60–80)	105 (100–120)	100 (98–105)	78 (60–80)
Proteinuria (g/l)	0 (0–0.3)	3 (0.3–5)	3 (0.3–5)	0 (0-1)
Birth weight (g)^a^	3780 (2900–4315)	3100 (2500–3860)	1590 (1100–3430)	2790 (1400–3850)
Gender M : F	7 : 8	5 : 5	2 : 3	3 : 2
Pulsatility index	NA	0.69 (0.59–0.73)	0.99 (0.67–1.43)	0.93 (0.83–1.76)
Mode of delivery (VD : CS)	15 : 0	7 : 3	3 : 2	4 : 1

All data is presented as median (range) except child gender (Male : Female) and mode of delivery (VD : CS). A Kruskal-Wallis post hoc Dunn test was used to calculate statistical differences between the groups. PE: pre-eclampsia, PE/N: pre-eclampsia with bilateral notching, NA: not available, VD: vaginal delivery, and CS: caesarian section. ^a^A significant difference between controls versus PE/N (*P* < .05).

**Table 2:** The most significantly altered genes in the microarray experiments, with their respective gene ontology classification (where available).

Gene symbol	Genebank	*P*-value	Gene ontology	FC
PE compared to control				
*C1orf90	NM_032648	7.0 × 10^−5^		1.9
*TGFB1	NM_000660	.0011	Cell death, inflammatory response, negative regulation of cell proliferation, and so forth	3.5
*INHA	NM_002191	.0014	Cytokine activity, hormone activity, hemoglobin biosynthetic process, cell-cell signaling, and so forth	2.3
* FBN2*	NM_001999	.0015	Extracellular matrix structural constituent, proteinaceous extracellular matrix	−2.2
* HOXA13*	NM_000522	.0027	Skeletal development hematopoiesis	2.1
* FLT1*	NM_002019	.03	Vascular endothelial growth factor receptor activity and positive regulation of cell proliferation	2.4

PE compared to notch				
*NCAM1	NM_000615	3.9 × 10^−4^	Cell adhesion, plasma membrane, synaptic transmission	1.7
*TIP30	NM_006410	.00048	Regulation of angiogenesis, regulation of apoptosis, RNA polymerase II transcription factor activity, and so forth	2.2
* CCL8*	NM_005623	.00060	Chemokine activity, calcium ion transport, chemotaxis, signal transduction, and so forth	3.4
* LY6D*	NM_003695	.00091	Cell adhesion	3.3
* ATP2A2*	NM_001681	.0013	Calcium transporting ATPase activity, cell adhesion, integral to plasma membranes, and so forth	−2.4
* SMARCA5*	NM_003601	.0015	RNA polymerase II transcription factor activity, DNA binding, transcription initiation, and so forth.	2.4

PE with notch compared to notch				
* KLHL31*	NM_001003760	.0019		1.9
* SPP1 *	NM_000582	.0020	Cell adhesion, and so forth	−2.8
* TBCD*	NM_005993	.0047	Chaperon binding	−1.9
* CIDEA *	NM_001279	.0055	Cell death, apoptotic, program, and so forth	−1.8

Notch compared to controls				
* TUBG1*	NM_001070	.0020	Structural constituent of cytoskeleton, protein binding, and so forth	−3.4
* GAL3ST4*	NM_024637	.0022	Cell-cell signaling, proteoglycan biosynthetic process, and so forth	−2.7
* CD320*	NM_016579	.0027	Regulation of cell growth	−3.7
*HP	NM_005143	.0061	Cellular iron ion homeostasis and defense response	−3.8

FC: fold change. All FC values are relative to the first group in the comparison. PE: pre-eclampsia

*Gene validated with quantitative real-time PCR.

**Table 3:** GO categories. All genes on the array were assigned to their respective GO categories and used as a background for the analysis.

GO Term	GO ID	*P*-value	FC
PE compared to controls			
Transcription factor binding	GO:0008134	.0019	3.6
* JUNB, CALCOCO1, ATF5, HOXC6, PARD6A, NR2F2, FAF1*			
Negative regulation of signal transduction	GO:0009968	.0027	8.5
*FRZB, ZA20D1, *and* RHOH *			
Transcription corepressor activity	GO:0003714	.0086	6.2
* JUNB, ATF5, HOXC6, *and* NR2F2 *			
Steroid hormone receptor activity	GO:0003707	.014	7.8
* PGRMC2, NR2F2, NR1D2*			

PE compared to notch			
Transcriptional activator activity	GO:0016563	.0005	3.2
*CALCOCO1, SMARCC1, PIAS1, SRCAP, RUNX1, MAML3, *and* DYRK1B *			
Muscle fiber development	GO:0048747	.0058	10.6
*IGFBP3, *and* CACNA1H *			
Cell motility	GO:0006928	.012	2.4
*FLNA, FEZ2, CRK, MAPK14, PXN, *and* TLN1 *			
Positive regulation of transcription, DNA-dependent	GO:0045893	.012	3.6
*PIAS1, SMARCC1, RUNX1, MAML3 *and* DYRK1B *			
Glucosamine metabolism,	GO:0006041	.018	7.1
*CTBS *and* NAGK *			

PE with notch compared to notch			
Chemotaxis / Locomotory behavior	GO:0006935	.021	6.7
*CCBP2, SPP1, *and* CMKLR1 *			
Positive regulation of cell proliferation	GO:0008284	.026	6.1
*SPP1 *and* EBI3 *			
Vesicle-mediated transport	GO:0016192	.029	6.7
*ERGIC1, ZFYVE1, *and* RABGEF1 *			
Cytokine binding	GO:0019955	.048	8.4
*CCBP2, EBI3, *and* CMKLR1 *			

Notch compared to control			
Regulation of apoptosis	GO:0042981	.017	3.9
*DIABLO, IGFBP3, STAT1 *			
Sterol transport	GO:0015918	.021	9.3
*NPC1 *and* CAV1 *			

PE: pre-eclampsia, FC: fold change, GO ID: gene ontology identification.

**Table 4:** Results from the KEGG signaling pathway analyses and the InterPro protein domain analyses.

KEGG pathway analyses	KEGG ID	*P*-value	FC
Pathway
PE compared to controls			
Neurodegenerative disorders	HSA01510	.04	9.1
*SOD1 *and* PSEN1 *			
PE without notch compared to notch			
Antigen processing and presentation	HSA04612	.04	3.1
*HLA-B, HSP90AA1, PSME2, *and* CD74 *			
PE with notch compared to notch			
Ubiquitin mediated proteolysis	HSA04120	.02	12.0
*UBE2E2, TCEB2, *and* ANAPC2 *			
Notch compared to controls			
Leukocyte transendothelial migration	HSA04670	.03	5.7
*ACTG1, FKSG30, CXCR4, *and* CLDN5 *			
Focal adhesion	HSA04510	.03	4.0
* ACTG1, FKSG30, FLT1, *and* CAV1 *			

InterPro protein domain analyses	InterPro ID	*P*-value	FC
InterPro term			

PE compared to controls			
HEAT	IPR000357	.05	4.5
*TNPO1 *and * PPP2R1A *			
Ras small GTPase, Rab type	IPR003579	.05	3.6
* RAB9B, RAB17, *and* RHOH *			
PE compared to notch			
HEAT	IPR000357	.003	4.3
* ARMC8, IPO13, PPP1R12B, *and* GCN1L1 *			
H+ transporting ATPase, proton pump	IPR000695	.008	21.4
* ATP13A1 *and* ATP2A2 *			
Haloacid dehalogenase-like hydrolase	IPR005834	.01	5.7
* ATP13A2, ATP13A1, *and * ATP2A2 *			
PE with notch compared to notch			
ATPase, F0/V0 complex	IPR002379	.02	7.4
* ATP6V0C *and* ATP5G1 *			
Notch compared to controls			
Actin	IPR004001	.04	44.1
* ACTG1 *and* FKSG30 *			

PE: pre-eclampsia, FC: fold change.
