# Supplementary material for: Differential Gene Expression Analysis of Placentas with Increased Vascular Resistance and Pre-Eclampsia Using Whole-Genome Microarrays
Source: J Pregnancy. 2011 Mar 8;2011:472354. doi: 10.1155/2011/472354 (PMC3066560; doi:10.1155/2011/472354)
Supplement: Supplementary file 1 — Supplementary table 1. Gene expression results from the microarray analysis. A matrix showing all genes that were found to be significantly altered. Results are presented as P-value (Fold change). Genes with a p-value<0.005 and FC>1.5 were considered statistically significant. PE=pre-eclampsia, N=bilateral notching without pre-clampsia and PEwN=preeclampsia with bilateral notching. [file 472354.f1.pdf]

Table legend for the supplementary table:

Supplementary table 1. Gene expression results from the microarray analysis. A matrix showing all genes that were found to be significantly altered. Results are presented as P-value (Fold change). Genes with a p-value < 0.005 and FC > 1.5 were considered statistically significant. PE = pre-eclampsia, N = bilateral notching without pre-clampsia and PEwN = pre-eclampsia with bilateral notching.

Gene symbol	Gene ID	PE vs. C	N vs. C	PE vs. N	PEwN vs. N
ACTG1	NM_001614		0.0047 (2.4)		
ADRB1	NM_000684				0.0049 (1.6)
AGR3	NM_176813				0.0043 (2.6)
AHDC1	NM_001029882			0.00079 (2.6)	
AMD1	NM_001634			0.00082 (-1.9)	
ANAPC2	NM_013366				0.0043 (2.1)
ANKRD10	NM_017664			0.0044 (-2.3)	
ARMC8	NM_015396			0.0030 (2.9)	
ARMET	NM_006010			0.0046 (-2.4)	
ATF5	NM_012068	0.0027 (1.6)			
ATP13A1	NM_020410			0.0050 (-2.9)	
ATP13A2	NM_022089			0.0044 (-2.5)	
ATP2A2	NM_001681			0.0013 (-2.4)	
ATP5G1	NM_005175				0.0050 (2.1)
ATP6V0C	NM_001694				0.0044 (-2.7)
ATRNL1	NM_207303			0.0042 (2.1)	
BBC3	NM_014417	0.0016 (1.9)			
C11orf72	NM_173578		0.0046 (3.9)		
C19orf29	NM_021231	0.0044 (2.0)			
C19orf56	NM_016145				0.0042 (-1.7)
C1orf90	NM_032648	7.0×10-5 (1.9)		0.00047 (2.8)	
C20orf11	NM_144629			0.0036 (2.2)	
CACNA1H	NM_021098			0.0041 (2.3)	
CALCOCO1	NM_020898	0.0047 (1.8)		0.0050 (2.7)	
CAV1	NM_001753		0.0045 (3.8)		
CCBP2	NM_001296				0.0043 (1.9)
CCL8	NM_000060			0.00060 (3.4)	
CD320	NM_016579		0.0027 (-3.7)		
CD74	NM_004355			0.0041 (-6.8)	
CDK2AP2	NM_005851			0.0011 (2.8)	
CDON	NM_016952	0.0019 (-1.9)		0.0033 (-2.6)	
CEL	NM_001807				0.0029 (1.7)
CIB1	NM_006384		0.0050 (3.9)		0.0032 (2.4)
CIDEA	NM_001279				0.0055 (-1.8)
CLDN5	NM_003277		0.0050 (2.1)		
CMKLR1	NM_004072				0.0041 (1.7)
CRK	NM_016823			0.0033 (-2.1)	

CRYAB	NM_001885	0.0013 (-1.9)			
CTBS	NM_004388			0.0020 (2.5)	
CXCR4	NM_003467		0.0043 (-2.4)		
DAB2	NM_001343	0.0032 (-2.2)			
DDX48	NM_014740				0.0048 (-3.4)
DIABLO	NM_138929		0.0050 (-2.7)		
DOPEY1	NM_015018			0.0037 (2.8)	
DYRK1B	NM_004714			0.0050 (2.4)	
EBI3	NM_005755				0.0044 (3.3)
EDF1	NM_003792				0.0042 (-3.1)
ERGIC1	NM_020462				0.0045 (-2.2)
FAF1	NM_007051	0.0023 (-1.6)			
FBN2	NM_001999	0.0015 (-2.2)			
FEZ2	NM_005102	0.0039 (2.0)		0.0033 (2.3)	
FKSG30	XM_001723912		0.0050 (2.0)		
FLJ22746	NM_024785	0.00018 (2.3)			
FLNA	NM_001456			0.0044 (-2.5)	
FLT1	NM_002019	0.03 (2.4)	0.0050 (3.0)		
FUS	NM_004960			0.0046 (3.2)	
GAL3ST4	NM_024637		0.0022 (-2.7)		
GCN1L1	NM_006836			0.00048 (2.8)	
GTF2E2	NM_002095			0.0020 (2.6)	
HLAB	NM_005514			0.0041 (-2.9)	
HOXA13	NM_000522	0.0027 (2.1)			
HOXC6	NM_004503	0.0050 (2.8)			
HP	NM_005143		0.0061 (-3.8)		
HSD17B1	NM_000413	0.00046 (-2.4)		0.0014 (-2.3)	
HSP90AA1	NM_005348			0.0041 (-2.5)	
IGFBP3	NM_000598		0.0043 (2.9)	0.0048 (-2.8)	
INHA	NM_002191	0.0014 (2.3)			
IPO13	NM_014652			0.0041 (-2.8)	
JUNB	NM_002229	0.0047 (2.0)			
KIAA0404	NM_015104			0.0043 (2.1)	
KIAA1822	NM_032425	0.0016 (2.1)			
KLHL31	NM_001003760				0.0019 (-1.9)
LENG1	NM_024316				0.0044 (2.9)
LOC162632	NR_003190				0.0045 (1.9)
LPP	NM_005578	0.0033 (1.9)		0.0023 (2.2)	
LY6D	NM_003695			0.00091 (3.3)	
MAFF	NM_012323				0.0015 (-2.7)
MAML3	NM_018717			0.0048 (-1.8)	
MAP3K4	NM_005922			0.0049 (2.8)	
MAP4K2	NM_004579			0.0041 (2.9)	
MBD6	NM_052897	0.0050 (1.9)			
MEF2D	NM_005920			0.0043 (1.9)	
MGC10471	NM_030818		0.0046 (3.5)		

NAGK	NM_017567			0.0041 (-2.9)	
NCAM1	NM_000615	0.0046 (1.7)		0.00039 (2.6)	
NISCH	NM_007184			0.0042 (2.1)	
NPC1	NM_000271		0.0043 (-3.5)		
NR1D2	NM_005126	0.0037 (2.5)			
NR2F2	NM_021005	0.0023 (1.7)			
PAPPA2	NM_020318		0.0035 (3.1)		
PARD6A	NM_016948	0.0047 (1.9)			
PCBP4	NM_033008	0.00070 (1.7)			
PCTP	NM_021213			0.0030 (2.8)	
PDLIM7	NM_005451			0.0013 (1.7)	
PGRMC2	NM_006320	0.0020 (-1.8)			
PIAS1	NM_016166			0.0011 (3.3)	
PIK3C2B	NM_002646	0.0033 (2.0)			
PLCXD2	NM_153268			0.0016 (2.2)	
PMPCA	NM_015160		0.0050 (-2.7)		
PNKD	NM_000396			0.0019 (2.5)	
PNPLA5	NM_138814			0.0036 (1.8)	
POLDIP3	NM_178136			0.0024 (-2.3)	
PPP1R12B	NM_032105			0.0013 (2.8)	
PPP2R1A	NM_014225	0.0043 (-2.1)			
PSEN1	NM_000021	0.0027 (1.9)			
PSME2	NM_002818			0.0048 (-2.7)	
PXN	NM_002859			0.0049 (-3.0)	
RAB17	NM_022449	0.0050 (2.0)			
RAB9B	NM_016370	0.0044 (2.0)			
RABGEF1	NM_014504				0.0050 (-2.1)
RHOH	NM_004310	0.0027 (1.8)			
RUNX2	NM_004348			0.0046 (2.4)	
SANG	NM_002785			0.000019 (2.9)	
SCN4A	NM_000334				0.0016 (-1.7)
SH3PXD2A	NM_014631		0.0050 (3.4)		
SLC20A1	NM_005415	0.0047 (-2.2)			
SLC27A3	NM_024330	0.0011 (2.1)			
SLC39A7	NM_006979	0.0047 (1.7)			
SMARCA5	NM_003601			0.0015 (2.4)	
SMARCC1	NM_003074			0.0044 (-2.0)	
SOD1	NM_000454	0.0037 (-1.9)			
SPP1	NM_000582				0.0020 (-2.8)
SRCAP	NM_006662			0.0042 (-2.5)	
STAT1	NM_007315		0.0043 (2.2)		
TAF15	NM_003487	0.00047 (2.1)			
TBCD	NM_005993				0.0047 (-1.9)
TCEB2	NM_007108				0.0043 (2.9)
TGFB1	NM_000660	0.0011 (3.5)			
TGFBR1	NM_004612			0.0037 (-2.4)	

TIP30	NM_006410			0.00048 (2.2)	
TKT	NM_001064	0.0035 (-2.2)			
TLN1	NM_006289			0.0045 (-2.9)	
TMEM93	NM_031298			0.0043 (2.2)	
TNPO1	NM_002270	0.0047 (-2.0)			
TRPM2	NM_003307			0.0027 (-2.3)	
TUBG1	NM_001070		0.0020 (-3.4)		
UBA52	NM_003333			0.0028 (-1.9)	
UBE2E2	NM_152653				0.0043 (-2.5)
ULK4	NM_017886	0.0049 (1.7)		0.0029 (2.5)	
WDR33	NM_018383	0.0013 (1.9)			
VKORC1L1	NM_173517			0.0043 (2.6)	
YIPF4	NM_032312	0.0048 (-1.8)			
ZA20D1	NM_020205	0.0029 (2.2)			
ZFP36	NM_003407			0.0035 (-3.5)	
ZFYVE1	NM_021260				0.0043 (-2.7)
ZNF16	NM_006958			0.0049 (2.5)	
ZNF549	NM_153263			0.0046 (-2.5)	
ZNF557	NM_024341	0.0016 (1.9)			
